# *Limosilactobacillus fermentum* IOB802 Protects Against Blue Light-Induced Retinopathy via Gut Microbiota Modulation

**DOI:** 10.3390/nu17223517

**Published:** 2025-11-11

**Authors:** Chen Liu, Yuqi Zhao, Jia Li, Shiqi Gao, Jin Cao, Na Jing, Xuemei Han, Hongpeng He, Wu Liang, Nan Wang

**Affiliations:** 1 College of Biotechnology, Tianjin University of Science and Technology, Tianjin 300457, China; liuchenstc@163.com (C.L.); 19957205857@163.com (Y.Z.); 18763702327@163.com (J.L.); gsq143680946@163.com (S.G.); kindloveless@163.com (J.C.); 18364402587@163.com (N.J.); hehongpeng@tust.edu.cn (H.H.); 2 Tianjin Key Laboratory of Edible Probiotics, Tianjin 300301, China; hanxuemei2024@163.com

**Keywords:** antioxidant, blue light, gut microbiota, retina

## Abstract

Background: Blue light-induced retinal photodamage represents a growing public health concern globally. Lactic acid bacteria and their bioactive metabolites represent a promising therapeutic strategy for mitigating such damage. Methods: This study evaluated the protective efficacy of *Limosilactobacillus fermentum* IOB802 and *Lactobacillus plantarum* subsp. *plantarum* IOB602 against blue light-induced retinal injury using both in vitro and in vivo models. Results: In ARPE-19 cells exposed to blue light, treatment with postbiotics from IOB802 and IOB602 significantly restored cell viability (*p* < 0.05), enhanced antioxidant enzyme activities (GSH-Px, SOD, and CAT, *p* < 0.05), and reduced inflammatory cytokine levels (IL-6, IL-1β, TNF-α, and VEGF, *p* < 0.05). Subsequent validation in a murine blue light-induced retinal damage model demonstrated that IOB802 notably preserved retinal architecture, upregulated antioxidant defenses, and promoted the expression of tight junction proteins. Mechanistically, IOB802 suppressed inflammation by inhibiting the phosphorylation of the IκBα/NF-κB pathway. Through 16S rDNA sequencing and short-chain fatty acid (SCFA) profiling, IOB802 was further shown to restore gut microbial diversity, increase beneficial bacteria, including *Lachnospiraceae*, *Rikenellaceae*, and *Bacteroidaceae* (*p* < 0.05), and elevate concentrations of key SCFAs (butyrate, acetate, and propionate; *p* < 0.05), underscoring the role of the gut–retina axis in mediating retinal protection. Conclusions: In summary, IOB802 and its postbiotics alleviate blue light-induced retinopathy through antioxidative, anti-inflammatory, and microbiota-modulating mechanisms, offering novel insights into microbiome-based interventions for retinal diseases.

## 1. Introduction

Asthenopia (blue light-induced retinal dysfunction), a multifactorial syndrome resulting from chronic stress-induced damage to the visual system, was clinically characterized by ocular pain, diminished visual quality, and concomitant headaches [1,2]. With the exponential increase in electronic device usage, this condition emerged as a global public health concern, particularly among adolescent populations. Epidemiological data revealed that 53.3% of adolescents reported eye fatigue, 38.9% exhibited blurred vision (manifested as impaired near-far focal accommodation), and 34.2% experienced ocular irritation or burning sensations following prolonged digital exposure [3]. The progression of this pathological state demonstrated significant correlation with degenerative retinal functional alterations, where the oxidative stress-inflammatory cascade was identified as the central pathogenic driver. As the most metabolically active neural tissue, retinal homeostasis critically depended on the synergistic interplay between the retinal pigment epithelium (RPE) and neurosensory layers. Substantial evidence indicated that when endogenous antioxidant systems (superoxide dismutase and catalase) failed to counteract reactive oxygen species (ROS) overproduction, cellular redox imbalance triggered inflammatory cascades [4]. Mélanie et al. [5] established through blue light exposure models that RPE cells exhibited increased ROS levels (The level of hydrogen peroxide increased tenfold), accompanied by mitochondrial membrane potential collapse and respiratory chain inhibition, alongside decreases in SOD/CAT activities, confirming phototoxic disruption of retinal redox homeostasis. Notably, pathological ROS accumulation activated the NF-κB signaling pathway, promoting NLRP3 inflammasome assembly and subsequent release of pro-inflammatory cytokines, thereby perpetuating retinal injury cycles [6]. Current clinical interventions predominantly relied on artificial tears (containing vitamin A/C/E and zinc formulations) or lutein supplements [7]. While these approaches transiently improved ocular surface lubrication, they demonstrated limited efficacy in addressing deep retinal oxidative lesions or systemic inflammatory responses.

Recent years have witnessed the emergence of the “gut–eye axis” theory, which provided novel perspectives for understanding the mechanisms of asthenopia [8]. This theory emphasized that gut microbiota could influence ocular microenvironments through metabolite exchange, immune modulation, and neural signaling. Clinical studies revealed dysbiosis in glaucoma patients, characterized by an altered Firmicutes/Bacteroidetes ratio and increased abundance of the pro-inflammatory genus *Dysgonamonadaceae*, which significantly correlated with retinal neurodegenerative progression [9]. Morita et al. [10] demonstrated that *Lactobacillus paracasei* KW3110 could effectively suppress inflammatory responses and photoreceptor cell degeneration in light-induced retinopathy models by promoting macrophage M2 polarization. Kim et al. [11] demonstrated that the IRT-5 probiotic formulation alleviated experimental autoimmune uveitis by restoring Th17/Treg balance, highlighting the therapeutic potential of probiotic interventions in ocular diseases. Short-chain fatty acids (SCFAs), key metabolites derived from gut microbial fermentation of dietary fibers, were shown to regulate ocular immune responses and suppress inflammasome activation. Among probiotics, Lactobacillus emerged as a prominent candidate due to its robust environmental tolerance (acid and osmotic resistance) and multifunctional bioactivities [12]. Wang et al. [13] developed a multilayer microcapsule encapsulating *Lactobacillus rhamnosus* YBT20 and demonstrated that this formulation effectively alleviated oxidative damage in the rat retinal pigment epithelium while significantly suppressing the levels of inflammatory factors such as IL-6, IL-8, IL-1β, and TNF-α. Their findings provided an important theoretical basis for the targeted delivery of probiotics and retinal protection strategies. However, existing studies had not systematically elucidated the potential protective mechanisms of gut microbiota and their metabolites against retinal light-induced damage through modulating oxidative stress imbalance and related signaling pathways. Previous collaborative studies have shown that the fermented *Limosilactobacillus fermentum* IOB802 exhibited significant activity in skin antioxidant defense and bone metabolism regulation, while *Lactobacillus plantarum* subsp. *plantarum* IOB602 showed specific improvements in central nervous system function [14,15]. However, whether these two strains can regulate retinal oxidative stress and inflammatory responses via the gut–eye axis remains to be systematically studied.

Building on these findings, this study conducted the first systematic evaluation of the interventional effects of *L. fermentum* IOB802 and *L. plantarum* IOB602 in blue light-induced retinal injury models. Focusing on blue light-induced retinal pathological damage—which is recognized as an important objective pathological basis of clinical asthenopia syndrome—we established both an ARPE-19 cell oxidative damage model and a blue light-exposed murine retinopathy model. The research aimed to clarify the strain-specific retinoprotective effects of these probiotics, elucidate their mechanisms in modulating retinal dysfunction via the gut microbiota–SCFA metabolic axis, and delineate the molecular regulatory network involving the NF-κB signaling pathway. These findings provide a theoretical foundation for developing microbiome-targeted strategies against blue light-induced visual impairment.

## 2. Materials and Methods

### 2.1. Probiotic Strains and Postbiotic Preparation

The experimental strains *Limosilactobacillus fermentum* IOB802 (CGMCC No. 23120) and *Lactobacillus plantarum* subsp. *plantarum* IOB602 (CGMCC No. 16021) were obtained from Tianjin Chuangyuan Biotechnology Co., Ltd., Tianjin, China. IOB802 was isolated from traditional fermented kimchi, while IOB602 originated from naturally fermented sourdough collected from households in Tianjin, China. Both strains were authenticated via 16S rRNA gene sequencing and deposited at the China General Microbiological Culture Collection Center (CGMCC; No. 3, Yard 1, Beichen West Road, Chaoyang District, Beijing, China).

Strains were cultured anaerobically in de Man, Rogosa and Sharpe (MRS) broth at 37 °C for 22 h. Bacterial cells were harvested by centrifugation, washed twice, and resuspended in sterile phosphate-buffered saline (PBS) to a final concentration of 1 × 10^9^ CFU/mL. Postbiotics were prepared by thermal inactivation (90 °C, 15 min) of washed cell pellets, followed by lyophilization. The resulting postbiotic preparations contained inactivated cells and residual MRS components. To control for medium interference, an MRS vehicle control (Veh group) was included. All strains underwent biosafety validation by PONY Testing International Group Co., Ltd., Beijing, China, including assessments of hemolytic activity, antibiotic resistance genes, and acute oral toxicity.

### 2.2. Establishment of Blue Light-Induced Damage Model in ARPE-19 Cells

The human retinal pigment epithelial cell line ARPE-19 (CL-0026, Procell Life Science & Technology Co., Ltd., Wuhan, China) was cultured in specialized medium (CM-0026, Procell) supplemented with 10% fetal bovine serum (FBS) and 1% penicillin-streptomycin, maintained at 37 °C under 5% CO_2_. A blue light exposure model was established based on a modified protocol from reference [10,16]: cells were irradiated using a 460 nm monochromatic LED array (illuminance 2500 ± 200 Lux) with intensity calibrated at the culture plate surface using a spectroradiometer (LS-160, Konica Minolta, Tokyo, Japan).

At 60–70% confluency, cells were pretreated with IOB802/IOB602 postbiotics (1 × 10^4^–1 × 10^8^ CFU/mL) for 6 h, while the model control group (MC) received basal medium. Following pretreatment, cells underwent 12 h of blue light exposure. Cell viability was assessed via MTT assay (Sigma-Aldrich, St. Louis, MO, USA). Post-exposure, culture supernatants were collected, and cells were ultrasonically lysed. Lysates were centrifuged (12,000× *g*, 15 min, 4 °C), with clarified supernatants stored at −80 °C for subsequent analysis.

### 2.3. Animal Experiment

SPF-grade 5-week-old male BALB/c mice (20 ± 2 g) were purchased from Spebio (Beijing, China) Biotechnology Co., Ltd. (Beijing, China, License No. SCXK (Jing) 2019-0010). All animal experiments were conducted in accordance with the Guide for the Care and Use of Laboratory Animals (NIH Publication No. 8023) and approved by the Animal Experiment Ethics Committee of Tianjin University of Science and Technology (No. SWXY-20230707520). Mice were housed in a barrier environment at Tianjin University of Science and Technology, with environmental parameters set to constant temperature (20–26 °C), relative humidity (40–70%), and a 12 h light/dark cycle, with free access to standard chow and sterilized water.

A randomized group design was employed (n = 8 per group), with a total of 8 groups. The groups were as follows: Normal Control (NC) group: saline gavage, no blue light exposure; Positive Control (PC) group: 10 mg/kg lutein solution gavage + blue light exposure; Model Control (MC) group: saline gavage + blue light exposure; IOB802 group: 10^8^ CFU/mL *Limosilactobacillus fermentum* IOB802 suspension gavage + blue light exposure; IOB602 group: 10^8^ CFU/mL *Lactobacillus plantarum* subsp. *plantarum* IOB602 suspension gavage + blue light exposure; MRS medium group: equal volume of MRS medium gavage; Pos-IOB802 group: 10^8^ CFU/mL IOB802 postbiotic preparation gavage + blue light exposure; Pos-IOB602 group: 10^8^ CFU/mL IOB602 postbiotic preparation gavage + blue light exposure. The intervention period lasted for 4 weeks, and all groups, except the NC group, received blue light exposure during the final week (5000 ± 500 Lux, 12 h/day for 7 days) [10]. During the experiment, food and water intake were monitored daily, and body weight changes were recorded weekly. At the end of the experiment, animals were euthanized by cervical dislocation, and serum, cecal contents, and retinal tissues were collected and stored at −80 °C for subsequent analysis. Animals in this study were randomly assigned using a random number table method, with blinding procedures implemented during all behavioral tests, histological evaluations, and data analysis phases regarding group assignment.

### 2.4. Oxidative Stress Marker Analysis

Antioxidant enzyme activities and lipid peroxidation levels in cell and retinal tissue samples were determined using commercial assay kits for catalase (CAT, BC0205), glutathione peroxidase (GSH-Px, BC1175), superoxide dismutase (SOD, BC0175), and malondialdehyde (MDA, BC0025). All kits were purchased from Nanjing Jiancheng Bioengineering Institute (Nanjing, China), and procedures were performed strictly following the manufacturer’s protocols.

### 2.5. Inflammatory Cytokine Analysis

Levels of inflammatory cytokines in cell and retinal samples were measured using enzyme-linked immunosorbent assay (ELISA) kits for interleukin-6 (IL-6, ZC-50163), interleukin-1β (IL-1β, ZC-37974), tumor necrosis factor-α (TNF-α, ZC-39024), and vascular endothelial growth factor (VEGF, ZC-35248). All ELISA kits were purchased from ZCIBIO Technology Co., Ltd. (Shanghai, China), and procedures were performed strictly following the manufacturer’s instructions.

### 2.6. Retinal Apoptosis Detection by TUNEL Assay

Retinal paraffin sections were deparaffinized in xylene, rehydrated through a graded ethanol series, and subjected to terminal deoxynucleotidyl transferase-mediated dUTP nick-end labeling using a TUNEL Apoptosis Detection Kit (C1090, Beyotime Biotechnology Co., Ltd., Shanghai, China). Procedures were performed as per the manufacturer’s protocol: sections were treated with Proteinase K (20 μg/mL) at 37 °C for 15 min, incubated with TUNEL reaction mixture (37 °C, light-protected, 1 h), and counterstained with DAPI (S2135, Solarbio Bioscience & Technology Co., Ltd., Beijing, China) for 5 min to label nuclei. Stained sections were imaged under a confocal microscope (LSM 900, Zeiss, Jena, Germany).

### 2.7. qPCR Gene Expression Analysis

Total RNA from cells and retinal tissue was extracted using the TransZol Up Plus RNA Kit (ER501, TransGen Biotech Co., Ltd., Beijing, China), and RNA purity was verified using the Nanodrop 2000. Genomic DNA was removed and cDNA synthesis was performed using the TransScript^®^ Uni All-in-One First-Strand cDNA Synthesis SuperMix for qPCR (One-Step gDNA Removal) (AU341, TransGen Biotech Co., Ltd., Beijing, China), with 1 μg of RNA template. Quantitative PCR was conducted using the PerfectStart^®^ Green qPCR SuperMix (AQ601, TransGen Biotech Co., Ltd., Beijing, China). The thermal cycling conditions were as follows: 30 s at 95 °C for pre-denaturation, followed by 40 cycles of 5 s at 95 °C and 30 s at 60 °C. Primer sequences are shown in Table 1. Relative gene expression was calculated using the 2^−ΔΔCt^ method, with GAPDH as the internal control.

### 2.8. Western Blot Analysis

Cellular and retinal tissue samples were lysed in ice-cold RIPA buffer (R0010, Beijing Solarbio Science & Technology Co., Ltd., Beijing, China) and centrifuged (12,000× *g*, 15 min, 4 °C) to collect supernatants. Protein concentrations were quantified using a BCA assay kit (PC0020, Solarbio Bioscience & Technology Co., Ltd., Beijing, China). Equal protein amounts (20 μg/lane) were mixed with 5× SDS-PAGE loading buffer (P1016, Beijing Solarbio), denatured at 95 °C for 5 min, and separated on 10% SDS-PAGE gels under constant voltage (80 V initial, increased to 120 V). Proteins were transferred to nitrocellulose (NC) membranes via wet transfer (120 V, 90 min). Membranes were blocked with 5% skim milk in TBST for 1 h at room temperature. Primary antibodies against IκBα (SC-1643), p-IκBα (SC-8408), NF-κB p65 (SC-398442), p-NF-κB p65 (SC-166748), and β-actin (SC-47778) (all from Santa Cruz Biotechnology, Santa Cruz, CA, USA) were incubated overnight at 4 °C. After three TBST washes (10 min each), membranes were probed with IRDye 800CW Goat anti-Mouse IgG (H + L) (926-32210, Li-Cor Biosciences, Lincoln, NE, USA) for 1 h. Protein bands were visualized using an Odyssey CLx imaging system (Li-Cor) and quantified via grayscale analysis with ImageJ software 1.8.0.

### 2.9. 16S rRNA Gene Sequencing of Intestinal Microbiota

Cecal content samples were rapidly frozen in liquid nitrogen and then subjected to microbial genomic DNA extraction using the CTAB (cetyltrimethylammonium bromide) method. The V3-V4 hypervariable region of the bacterial 16S rRNA gene was amplified with specific primers 338F (5′-ACTCCTACGGGAGGCAGCAG-3′) and 806R (5′-GGACTACHVGGGTWTCTAAT-3′). The PCR reaction system (25 μL) contained 2×Taq Plus Master Mix, 10 μM of each primer, and 50 ng of DNA template. The amplification protocol consisted of: initial denaturation at 95 °C for 3 min; 30 cycles of denaturation at 95 °C for 30 s, annealing at 55 °C for 30 s, and extension at 72 °C for 45 s; followed by a final extension at 72 °C for 10 min. Amplified products were purified by 2% agarose gel electrophoresis and then subjected to paired-end 300 bp sequencing on the Illumina MiSeq platform (Illumina, San Diego, CA, USA) by Shanghai Majorbio Bio-pharm Technology Co., Ltd. Raw data were quality-filtered using Cutadapt 3.5, followed by denoising and amplicon sequence variant (ASV) clustering with DADA2 1.24.0. Microbiota differential analysis was performed using the linear discriminant analysis effect size (LEfSe) method with a threshold of LDA score > 3.0 and *p* < 0.05. α-diversity and β-diversity analyses were conducted via the QIIME2 2022.2 platform.

### 2.10. Short-Chain Fatty Acids (SCFAs) Analysis

Fecal samples (0.1 g, accurately weighed) were homogenized in pre-cooled phosphate-buffered saline (PBS, pH 6.8), then centrifuged at 10,000× *g* for 5 min at 4 °C. One milliliter of the supernatant was mixed with 0.25 mL of 15% sulfuric acid-methanol solution (*v*/*v*) by vortexing, followed by derivatization at 60 °C for 30 min. The derivatized products were filtered through a 0.22 μm organic membrane and analyzed using gas chromatography with a flame ionization detector (GC-FID, Agilent 7890B, Santa Clara, CA, USA) equipped with an RB-624 capillary column (30 m × 0.32 mm × 1.8 μm, Agilent J&W). The analytical conditions were set as follows: inlet temperature 200 °C, split ratio 20:1; column temperature program starting at 60 °C for 3 min, then increasing at 10 °C/min to 80 °C; detector temperature 220 °C, hydrogen flow rate 30 mL/min, air flow rate 300 mL/min, and nitrogen (purity ≥ 99.999%) as the carrier gas at 1.5 mL/min. Headspace sampling parameters included: equilibrium temperature 80 °C (30 min), transfer line temperature 90 °C, quantitative loop temperature 100 °C, and injection volume 1 μL. Quantification was performed using the external standard method, with SCFA concentrations calculated from standard curves and expressed as μmol/g wet weight.

### 2.11. Data Analysis

All experimental data were presented as mean ± standard deviation. The normality of distribution for all datasets was assessed using the Shapiro–Wilk test, and homogeneity of variances was verified by Bartlett’s test. Based on these evaluations, appropriate statistical methods were selected. For data meeting normality assumptions, parametric tests were employed, including independent *t*-tests for two-group comparisons and one-way ANOVA with Tukey’s post hoc test for multi-group comparisons. For non-normally distributed data, particularly microbiota relative abundance data, non-parametric tests were applied using Mann–Whitney U tests for two-group comparisons and Kruskal–Wallis H tests with Dunn’s post hoc correction for multi-group comparisons. In microbiome 16S rRNA sequencing analyses, false discovery rate (FDR) correction was implemented to account for multiple comparisons. Effect sizes were calculated for all key comparisons, with Cohen’s d for parametric tests and r values for non-parametric tests, and reported alongside exact *p*-values and sample sizes in the figure legends. All statistical analyses and data visualizations were performed using GraphPad Prism 8 (Version 8.0.2, USA).

## 3. Results

### 3.1. IOB802 and IOB602 Mitigate Blue Light-Induced Retinal Damage via Oxidative and Inflammatory Pathways in ARPE-19 Cells

**Figure 1:**
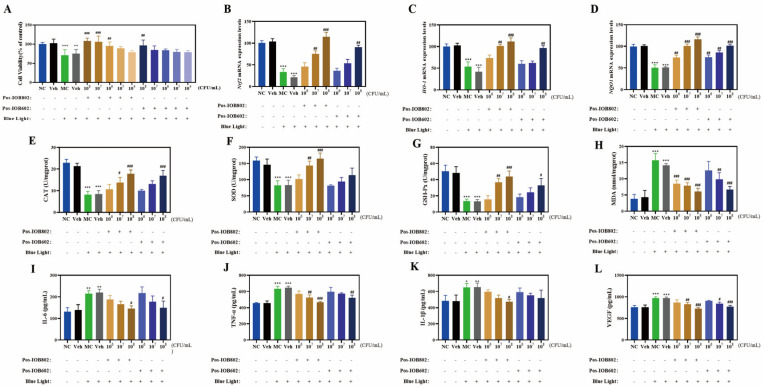
Protective effects of IOB802/IOB602 postbiotics against blue light-induced damage in ARPE-19 cells. (**A**) Cell viability of ARPE-19 cells treated with Pos-IOB802 and Pos-IOB602 (10^4^–10^8^ CFU/mL) was determined by MTT assay. (**B**–**D**) The mRNA expression levels of Nrf2 (**B**), HO-1 (**C**), and NQO1 (**D**) were measured using qRT-PCR (n = 3). (**E**–**H**) The enzymatic activities of CAT, SOD, GSH-Px, and the content of MDA were assessed in ARPE-19 cells (n = 3). (**I**–**L**) The cytokine levels of IL-6, TNF-α, IL-1β, and VEGF were measured in ARPE-19 cells (n = 3). Data were analyzed by one-way ANOVA with Tukey’s multiple-comparison test. * *p* < 0.05, ** *p* < 0.01, *** *p* < 0.001 vs. NC group; # *p* < 0.05, ## *p* < 0.01, ### *p* < 0.001 vs. MC group.

To investigate the protective effects of probiotics IOB802 and IOB602 against blue light-induced damage at the cellular level, this study utilized ARPE-19 cells to evaluate their impacts on oxidative stress markers, inflammatory cytokines, and oxidative stress-related gene expression following blue light exposure. As illustrated in Figure 1A, both IOB802 and IOB602 effectively restored the reduction in cell viability caused by blue light exposure, with IOB802 demonstrating significant efficacy at concentrations of 10^8^, 10^7^, and 10^6^ CFU/mL. Consequently, these concentrations were selected for subsequent experiments.

In terms of oxidative stress markers (Figure 1E–H), the activities of CAT, SOD, and GSH-Px in the MC group were significantly reduced to 35.94%, 51.79%, and 26.36% of the NC group, respectively, after blue light exposure. Concurrently, MDA levels increased 4.1-fold compared to the NC group. Treatment with IOB802 significantly restored CAT, SOD, and GSH-Px activities while reducing MDA content (*p* < 0.05, *p* < 0.01, *p* < 0.001). Notably, IOB802 exhibited pronounced protective effects at 10^8^ and 10^7^ CFU/mL, whereas IOB602 showed limited efficacy. Further analysis of oxidative stress-related genes revealed that IOB802 significantly upregulated the expression of Nrf2, HO-1, and NQO1 across all tested concentrations, while IOB602 only marginally elevated these genes at 10^8^ CFU/mL (Figure 1B–D). Regarding inflammatory cytokine expression (Figure 1I–L), the MC group displayed significantly elevated levels of IL-6, TNF-α, IL-1β, and VEGF compared to the NC group. Both IOB802 and IOB602 at 10^8^ CFU/mL markedly suppressed the expression of these cytokines, with IOB802 exhibiting stronger inhibitory effects. Specifically, IOB802 at 10^8^ CFU/mL reduced IL-6, TNF-α, IL-1β, and VEGF levels by 31.94%, 27.14%, 26.81%, and 25.21%, respectively. No significant differences were observed between the Veh group and the MC group (*p* > 0.05). These findings demonstrate that IOB802 confers effective cellular protection against blue light-induced damage by modulating oxidative stress and inflammatory responses, with concentration-dependent efficacy observed, particularly at higher concentrations.

### 3.2. Protective Effects of IOB802 and IOB602 Against Blue Light-Induced Retinal Damage in Mice

**Figure 2:**
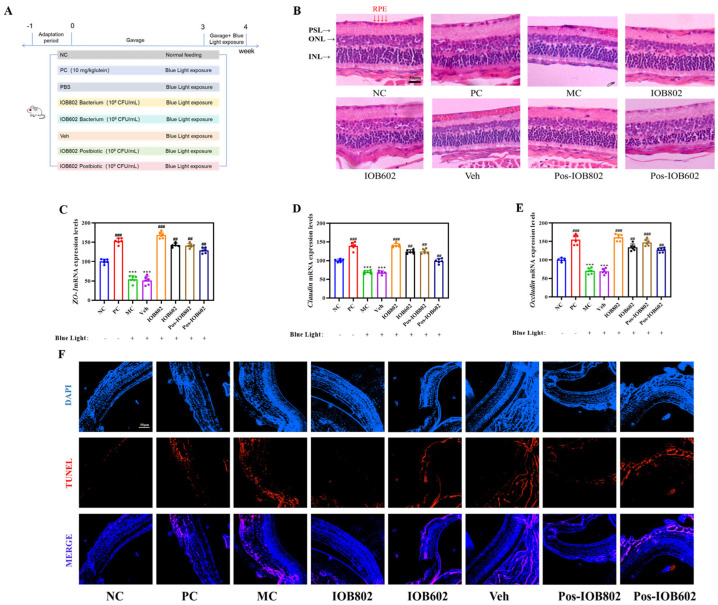
Protective effects of IOB802, IOB602, and their postbiotics against blue light-induced retinal damage in mice. (**A**) Schematic illustration of the experimental groups and treatment protocol administered to the mice. (**B**) Representative images of retinal morphology assessed by H&E staining (scale bar = 50 μm). (**C**–**E**) mRNA expression levels of tight junction proteins ZO-1 (**C**), Claudin (**D**), and Occludin (**E**) in mice, measured by qRT-PCR (n = 6). (**F**) Apoptosis in retinal sections evaluated by TUNEL staining (scale bar = 50 μm). Data were analyzed by one-way ANOVA with Tukey’s multiple-comparison test. *** *p* < 0.001 vs. NC group; ## *p* < 0.01, ### *p* < 0.001 vs. MC group.

The protective effects of IOB802 and IOB602 were further evaluated through animal experiments in blue light-exposed mice (experimental design shown in Figure 2A). Histopathological assessment of retinal status revealed severe structural alterations in the MC group, including disorganized cellular arrangement, widened intercellular spaces, chromatin condensation, and reduced retinal layer organization. Both IOB802 and IOB602 treatments partially restored retinal morphology, with IOB802 demonstrating superior efficacy. The IOB802 group exhibited near-normal retinal stratification, tightly packed cellular architecture, and RPE cell density comparable to the NC group, indicating optimal protection (Figure 2B). Quantitative PCR analysis showed significantly downregulated expression of retinal structural protein genes (ZO-1, Claudin, and Occludin) in the MC group (*p* < 0.001). Both probiotics restored the expression of these genes, with IOB802 showing markedly stronger effects (Figure 2C–E). TUNEL staining further confirmed that IOB802 significantly suppressed blue light-induced retinal apoptosis (Figure 2F). These results demonstrate that IOB802 and IOB602 confer protection against blue light-induced retinal damage in mice, with IOB802 exhibiting substantially greater therapeutic efficacy.

### 3.3. Effects of IOB802 and IOB602 on Inflammation and Oxidative Stress in Mice with Blue Light-Induced Retinal Damage

**Figure 3:**
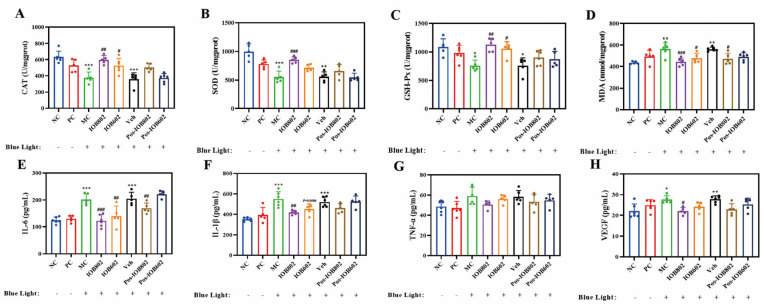
Effects of IOB802, IOB602, and their postbiotics on oxidative stress homeostasis and inflammatory factors in mice with blue light-induced retinal damage. (**A**–**D**) The enzymatic activities of CAT (**A**), SOD (**B**), GSH-Px (**C**), and the content of MDA (**D**) were measured in retinal tissue homogenates of mice (n ≥ 5). (**E**–**H**) The concentrations of pro-inflammatory and angiogenic cytokines, including IL-6 (**E**), IL-1β (**F**), TNF-α (**G**), and VEGF (**H**) in mice (n ≥ 5). Data were analyzed by one-way ANOVA with Tukey’s multiple-comparison test. * *p* < 0.05, ** *p* < 0.01, *** *p* < 0.001 vs. NC group; # *p* < 0.05, ## *p* < 0.01, ### *p* < 0.001 vs. MC group.

To further evaluate the regulatory effects of IOB802 and IOB602 on blue light-induced retinal inflammation and oxidative stress, serum biomarkers were analyzed. As shown in Figure 3A–D, blue light exposure significantly reduced serum CAT, SOD, and GSH-Px activities in the MC group to 59.55%, 55.19%, and 69.89% of the NC group, respectively, while MDA levels increased 1.26-fold. Both probiotics improved these parameters, with IOB802 demonstrating markedly stronger effects. Specifically, IOB802 treatment elevated CAT, SOD, and GSH-Px activities to 1.58-, 1.54-, and 1.48-fold of the MC group, respectively, and reduced MDA levels to 78.71% of the MC group.

Regarding inflammatory cytokines (Figure 3E–H), serum levels of IL-6, IL-1β, TNF-α, and VEGF in the MC group increased 1.56-, 1.56-, 1.22-, and 1.25-fold compared to the NC group. Both IOB802 and IOB602 significantly suppressed these inflammatory mediators, with IOB802 exhibiting superior efficacy. Notably, while TNF-α levels showed a decreasing trend following probiotic treatments, no statistically significant difference was observed compared to the MC group (*p* > 0.05). These data demonstrate that IOB802 confers retinal protection in blue light-exposed mice by systemically modulating redox homeostasis and inflammatory responses, with significantly greater efficacy than IOB602.

### 3.4. Modulation of NF-κB Signaling Pathway by IOB802 and IOB602

To investigate the regulatory effects of IOB802 and IOB602 on the NF-κB signaling pathway in blue light-induced retinal damage, phosphorylation levels of key signaling proteins were systematically evaluated via Western blot in ARPE-19 cells and C57BL/6 mouse retinal tissues.

**Figure 4:**
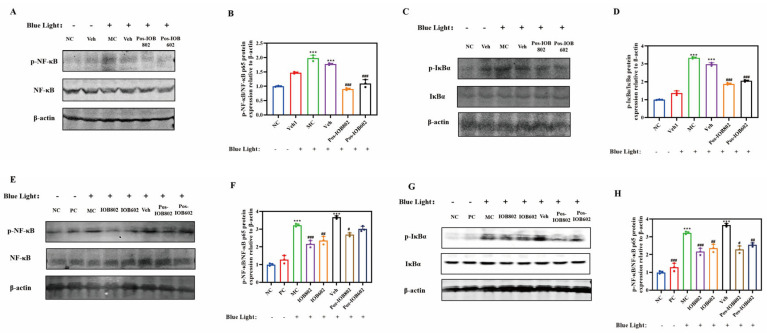
Effects of IOB802, IOB602, and their postbiotics on the NF-κB signaling pathway. (**A**,**B**) Representative immunoblots (**A**) and grayscale quantification (**B**) of p- NF-κB/NF-κB protein expression in ARPE-19 cells (n = 3); (**C**,**D**) Representative immunoblots (**C**) and grayscale quantification (**D**) of p-IκBα/IκBα protein expression in ARPE-19 cells (n = 3); (**E**,**F**) Representative immunoblots (**E**) and grayscale quantification (**F**) of p- NF-κB/NF-κB protein expression in mouse retinas (n = 3); (**G**,**H**) Representative immunoblots (**G**) and grayscale quantification (**H**) of p-IκBα/IκBα protein expression in mouse retinas (n = 3). Data were analyzed by one-way ANOVA with Tukey’s multiple-comparison test. *** *p* < 0.001 vs. NC group; # *p* < 0.05, ## *p* < 0.01, ### *p* < 0.001 vs. MC group.

In ARPE-19 cells (Figure 4A–D), the MC group exhibited significantly elevated ratios of phosphorylated IκBα to total IκBα (p-IκBα/IκBα) and phosphorylated NF-κB p65 to total NF-κB p65 (p-NF-κB/NF-κB) compared to the NC group (*p* < 0.001). Both IOB802 and IOB602 treatments markedly reduced p-IκBα and p-NF-κB phosphorylation levels, indicating suppression of IκBα degradation and subsequent inhibition of NF-κB p65 activation, thereby attenuating inflammatory responses. Consistent trends were observed in mouse retinal tissues (Figure 4E–H). The MC group showed significantly increased p-NF-κB p65 and p-IκBα protein expression compared to the NC group. All treatment groups (IOB802, IOB602, and their postbiotics) significantly reduced p-IκBα/IκBα and p-NF-κB/NF-κB ratios (*p* < 0.05, *p* < 0.01, *p* < 0.001). Notably, live bacterial formulations demonstrated superior anti-inflammatory efficacy compared to postbiotics, with IOB802 exhibiting the most pronounced inhibitory effects. These findings suggest that IOB802 and IOB602 mitigate blue light-induced retinal inflammation through NF-κB pathway modulation, with live IOB802 displaying optimal therapeutic potential.

### 3.5. Effects of IOB802 on Gut Microbiota Composition and Diversity in Mice with Blue Light-Induced Retinal Damage

**Figure 5:**
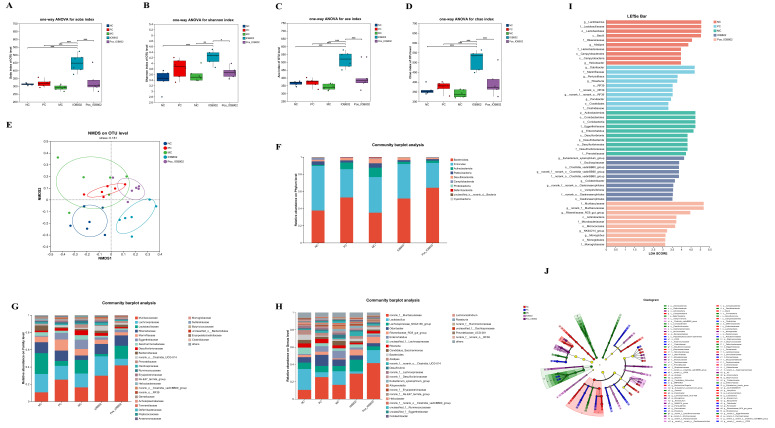
Effects of IOB802 and its postbiotics on the gut microbiota of mice with retinal damage. (**A**–**D**) Alpha diversity indices of the cecal microbiota as measured by the Sobs index (**A**), Shannon index (**B**), Ace index (**C**), and Chao index (**D**) (n = 6). (**E**) Beta diversity assessed by non-metric multidimensional scaling (NMDS) based on the Bray–Curtis distance. (**F**–**H**) Compositional profiling of gut microbiota at the phylum (**F**), family (**G**), and genus (**H**) levels. (**I**,**J**) Linear discriminant analysis Effect Size (LEfSe) analysis identifying differentially abundant taxa: bar plot showing LDA scores (**I**) and cladogram illustrating phylogenetic distribution of discriminative features (**J**). Data were analyzed by one-way ANOVA with Tukey’s multiple-comparison test. * *p* < 0.05, ** *p* < 0.01, *** *p* < 0.001 indicate statistically significant differences between groups.

**Figure 6:**
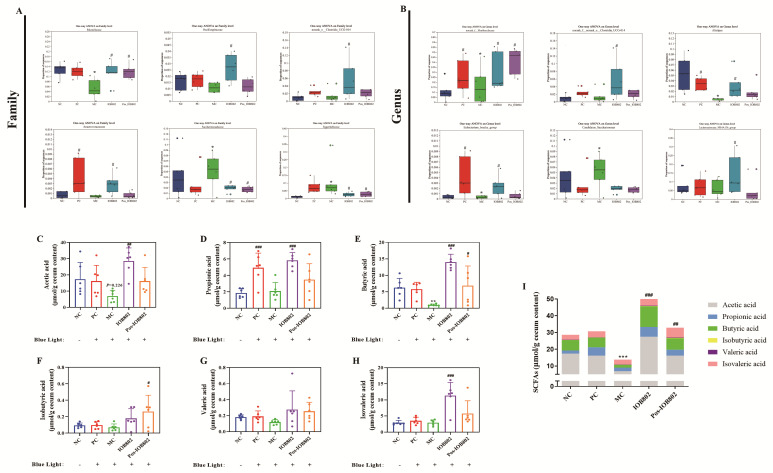
Effects of IOB802 and its postbiotics on gut microbiota and SCFAs in mice with retinal damage. (**A**,**B**) Comparative analysis of the relative abundances of specific microbiota at the family (**A**) and genus (**B**) levels (n = 6). (**C**–**H**) Fecal concentrations of acetic acid (**C**), propionic acid (**D**), butyric acid (**E**), isobutyric acid (**F**), valeric acid (**G**), and isovaleric acid (**H**) (n = 6). (**I**) Total SCFAs content in cecal feces (n = 6). Data were analyzed by one-way ANOVA with Tukey’s multiple-comparison test. * *p* < 0.05, ** *p* < 0.01, *** *p* < 0.001 vs. NC group; # *p* < 0.05, ## *p* < 0.01, ### *p* < 0.001 vs. MC group.

Given the superior neuroprotective effects of IOB802 observed in retinal photodamage models, its regulatory effects on gut microbiota structure were systematically analyzed using 16S rRNA sequencing. Alpha diversity analysis revealed that IOB802 significantly increased the Sobes, Chao, and ACE indices (*p* < 0.05), indicating enhanced microbial richness (Figure 5A–D). Concurrently, elevated Shannon indices (*p* < 0.05) reflected improved microbial diversity. Non-metric multidimensional scaling (NMDS) demonstrated distinct clustering patterns among the MC, NC, and IOB802 groups, suggesting effective reversal of blue light-induced gut dysbiosis by IOB802 (Figure 5E). At the phylum level, Bacteroidota, Firmicutes, and Actinobacteria dominated the gut microbiota across all groups. Family-level analysis showed reduced abundances of beneficial taxa, including *Lachnospiraceae*, *Rikenellaceae*, *Lactobacillaceae*, and *Bacteroidaceae*, in the MC group compared to the NC group. IOB802 intervention restored *Lachnospiraceae*, *Rikenellaceae*, and *Bacteroidaceae* abundances, whereas its postbiotic preparation (Post-IOB802) selectively enriched *Lactobacillaceae*. Notably, conditionally pathogenic families such as *Eggerthellaceae*, *Saccharimonadaceae*, *Desulfovibrionaceae*, *Prevotellaceae*, and *Ruminococcaceae* were significantly enriched in the MC group but reduced following IOB802 and Post-IOB802 treatments (Figure 5F–H). Genus-level profiling revealed that IOB802 reversed blue light-induced declines in *Lachnospiraceae*_NK4A136_group and *Rikenellaceae*_RC9_gut_group, while promoting the recovery of *Bacteroides* and *Alloprevotella* (Figure 5F–H). Post-IOB802 specifically enriched *Lactobacillus* and *Alloprevotella*. Additionally, IOB802 treatment normalized the aberrantly elevated abundances of *Enterorhabdus* and *Candidatus Saccharimonas* observed in the MC group. These results demonstrated consistent trends in the significant difference analysis at both the family and genus levels (Figure 6A,B). LEfSe analysis identified *Actinobacteriota*, *Coriobacteriales*, *Desulfovibrionia*, and *Eggerthellaceae* as biomarkers associated with retinal oxidative stress in the MC group. In contrast, IOB802-enriched taxa included core commensals such as *Eubacterium xylanophilum*_group, while Post-IOB802 further enhanced *Muribaculaceae* enrichment (Figure 5I–J).

### 3.6. Effects of IOB802 on Short-Chain Fatty Acid Metabolism in Mice with Blue Light-Induced Retinal Damage

To elucidate the regulatory effects of IOB802 and its postbiotic preparation on short-chain fatty acid (SCFA) metabolism in blue light-induced retinal damage, fecal SCFA profiles were analyzed. Compared to the NC group, the MC group exhibited significantly reduced concentrations of key functional SCFAs, including acetate, propionate, and butyrate. IOB802 intervention markedly restored these SCFA levels (*p* < 0.001), with superior efficacy compared to its postbiotic formulation (Post-IOB802). Additionally, isovalerate and valerate levels increased in both the IOB802- and Post-IOB802-treated groups. Total SCFA content analysis revealed that the IOB802 group showed significantly higher levels than the MC group and outperformed the Post-IOB802 group. These findings demonstrate that IOB802 treatment robustly enhances the production of acetate, propionate, and butyrate, restoring intestinal metabolic function and modulating gut homeostasis to attenuate inflammatory responses.

## 4. Discussion

Blue light (400–500 nm), the predominant emission spectrum of electronic devices, induces photooxidative damage to retinal pigment epithelial (RPE) cells through prolonged exposure. As a critical component of the outer retina, RPE cells exhibit high metabolic activity and oxygen consumption, rendering them particularly susceptible to ROS-mediated oxidative stress [17]. Studies have demonstrated that blue light triggers photodynamic effects in RPE cells, resulting in excessive production of hydrogen peroxide (H_2_O_2_) and superoxide anions (O_2_^−^), while significantly suppressing the activities of SOD and CAT [5,18]. Using the ARPE-19 cell model, Wu et al. [19] confirmed that blue light exposure elevates ROS levels, reduces cell viability, and increases apoptosis rates, processes closely associated with inhibition of the nuclear factor erythroid 2-related factor 2 (Nrf2) signaling pathway. Further investigations by Seol et al. [20] revealed that blue light-induced ARPE-19 damage correlates with increased nitric oxide (NO) generation and reduced SOD activity, whereas activation of Nrf2 phosphorylation effectively alleviates such oxidative injury.

Phosphorylation levels of key signaling proteins are widely recognized as critical molecular markers indicative of pathway activation status. This is particularly evident in the Nrf2 pathway, where Nrf2, a master regulator of oxidative stress responses, translocates to the nucleus upon ROS stimulation via phosphorylation, binds to antioxidant response elements (ARE), and drives the expression of phase II detoxifying enzymes, including NAD(P)H quinone oxidoreductase 1 (NQO1) and heme oxygenase-1 (HO-1) [21,22]. NQO1 prevents DNA oxidative damage by catalyzing two-electron reductions of quinones, while HO-1 scavenges free radicals through heme degradation, generating biliverdin and bilirubin [23,24]. Jun et al. [25] demonstrated that activation of the Nrf2/HO-1 pathway suppresses MDA accumulation, GSH depletion, and pro-inflammatory cytokine expression (IL-1β, IL-6, VEGF) in blue light-exposed RPE cells. This phosphorylation-based mechanism extends to other signaling pathways. In the study by Ruan et al. [26], adipocytes and triple-negative breast cancer cells demonstrated mutual activation through the CXCL1/IL-6-mediated STAT3/NF-κB p65 pathway, where elevated phosphorylation of both STAT3 and NF-κB p65 provided direct experimental evidence for pathway activation. Similarly, Zhang et al. [27] confirmed that Wendan Decoction specifically suppressed JAK2 and STAT3 phosphorylation, thereby effectively inhibiting the JAK/STAT signaling pathway activation. However, current research predominantly focuses on plant-derived extracts or synthetic compounds for retinal protection, leaving the mechanisms by which probiotics and their metabolites alleviate oxidative stress via the gut–retina axis largely unexplored. In this study, IOB802 and its postbiotics significantly upregulated Nrf2, HO-1, and NQO1 gene expression, enhanced CAT, SOD, and GSH-Px activities, and reduced MDA levels. Furthermore, they effectively suppressed the release of pro-inflammatory cytokines (IL-6, IL-1β, TNF-α, VEGF), with IOB802 demonstrating superior retinal protective effects compared to IOB602 in both cellular and animal models. These findings suggest that IOB802 may coordinately mitigate blue light-induced oxidative damage and inflammatory cascades by modulating the Nrf2/NF-κB signaling network through the gut microbiota–metabolite-immune axis, providing novel mechanistic insights for probiotic-based interventions in retinal degenerative diseases. To further elucidate transcriptional regulation mechanisms, subsequent studies would be conducted employing electrophoretic mobility shift assays and luciferase reporter gene assays, aiming to provide more direct evidence at the transcriptional level. This integrated approach provides novel mechanistic insights for probiotic-based interventions in retinal degenerative diseases.

Recent advances in the gut–retina axis theory have elucidated the intrinsic link between gut microbiota and retinal health. Studies have demonstrated that gut dysbiosis correlates with multiple ocular pathologies: Wang et al. [28] identified significant positive associations between elevated inflammatory mediators (IL-1β, TNF-α, CXCL10) and gut microbial imbalances in dry eye disease models, while Choi et al. [29] further confirmed that multi-strain probiotic interventions (e.g., *Lactobacillus casei*, *Lactobacillus acidophilus*) alleviate dry eye symptoms by restoring corneal barrier function and tear secretion. These findings suggest that probiotics may indirectly influence retinal pathophysiology via gut microbiome modulation, with short-chain fatty acids (SCFAs) serving as pivotal microbial metabolites in this process. For instance, Huang et al. [30] reported that sodium butyrate ameliorates diabetic retinopathy by enriching butyrate-producing taxa such as *Enterococcus*, *Lactobacillus*, *Bifidobacterium*, and *Lachnospiraceae_NK4A136_group*. Similarly, valproic acid suppresses pro-inflammatory cytokine expression (IFN-γ, IL-17, TNF-α, IL-1β) to mitigate retinal inflammation [31]. Chen et al. [32] demonstrated that short-chain fatty acids (SCFAs) suppress endotoxin-induced uveitis and inflammatory responses in retinal astrocytes. Accumulating evidence has established the protective effects of short-chain fatty acids (SCFAs) on retinal health. Previous studies demonstrated that SCFAs, characterized by their low molecular weight (<150 Da), water solubility, and weak acidity, were able to penetrate the blood-retinal barrier through passive diffusion and specific transporters such as monocarboxylate transporters [33,34]. This mechanism received experimental support from Chen et al. [32], who confirmed that SCFAs could cross the blood-retinal barrier via systemic circulation and modulate retinal immune responses, consistent with the trans-barrier transport mediated by monocarboxylate transporters. Through a systematic analysis of studies from the past decade, Ciurariu et al. [35] demonstrated that gut dysbiosis-induced alterations in SCFA metabolism were closely associated with exacerbated retinal inflammation, elevated oxidative stress, and vascular dysfunction. Notably, decreased SCFA levels were consistently correlated with the progression of various retinopathies, including diabetic retinopathy and age-related macular degeneration. Consequently, restoring SCFA homeostasis through dietary modifications or probiotic interventions emerged as a promising therapeutic strategy for retinal disorders. Building on these findings, subsequent investigations would focus on two primary objectives: exogenous SCFA administration and receptor blockade experiments to determine the direct contribution of SCFAs to retinal protection, coupled with comprehensive evaluation of blood-retinal barrier permeability to bacterial metabolites. This integrated approach would provide crucial evidence for elucidating the gut microbiota–SCFA–retina axis and advance our understanding of probiotic-mediated retinal protection through microbial metabolites.

In this study, IOB802 intervention significantly increased the abundances of SCFA-producing taxa, including *Rikenellaceae*, *Bacteroidaceae*, and *Alloprevotella* [36], while promoting the proliferation of butyrogenic genera such as *Lachnospiraceae_NK4A136_group* and *Eubacterium*. Notably, reduced *Lachnospiraceae* abundance has been strongly linked to elevated inflammatory cytokines [37], and IOB802 counteracted this by suppressing pathogenic taxa like *Eggerthellaceae* (associated with biamine-mediated mucosal injury [38]) and *Candidatus Saccharimonas* (positively correlated with IL-6 and TNF-α expression [39]), thereby restoring gut microbial equilibrium. This microbial restructuring directly enhanced SCFA synthesis (acetate, propionate, butyrate, and isovalerate). Mechanistically, acetate promotes regulatory T-cell differentiation via histone deacetylase (HDAC) inhibition, propionate suppresses Th17 inflammatory activity by inducing dendritic cell-derived IL-10, and butyrate inhibits the NF-κB pathway by downregulating NLRP3 inflammasome and GPR43 signaling [40,41]. NF-κB, a central hub of inflammatory regulation, is classically activated through IκB kinase-mediated phosphorylation of the p65 subunit [42]. Here, IOB802-induced SCFA elevation significantly suppressed p65 phosphorylation, reducing IL-6 and TNF-α release. This aligns with the “microbiota–metabolite-immune axis” proposed by Liu et al. [43] wherein probiotics enhance gut metabolic-immune homeostasis by modulating SCFA-producing taxa, ultimately alleviating blue light-induced retinal oxidative damage via NF-κB signaling. Unlike studies focusing on single SCFAs or synthetic compounds, IOB802 demonstrates multi-target, hierarchical regulatory advantages, offering novel mechanistic insights for probiotic-based interventions in retinal degenerative diseases. This study demonstrated that IOB802 and its postbiotics alleviated inflammation in blue light-exposed mice by modulating gut microbiota composition, enhancing SCFAs production, and suppressing the NF-κB signaling pathway. Building on existing research findings, subsequent investigations employed integrated metagenomic and metabolomic approaches to systematically elucidate the interaction mechanisms between dynamic gut microbiota alterations and short-chain fatty acid metabolic networks. The research team subsequently advanced the clinical translation process through sequential phases, including human tolerance assessment, formulation optimization, and cross-population efficacy validation, ultimately achieving systematic translation from fundamental research to clinical applications.

## 5. Conclusions

In summary, blue light-induced retinal damage primarily operates through oxidative stress and inflammatory mechanisms. The *Limosilactobacillus fermentum* IOB802 and its postbiotics demonstrated dual protective effects: they activated the Nrf2/HO-1 antioxidant pathway and suppressed NF-κB signaling, thereby enhancing endogenous antioxidant enzyme activity and mitigating retinal and RPE cell injury. Concurrently, these interventions modulated gut microbiota composition by promoting the proliferation of beneficial taxa such as *Rikenellaceae*, *Bacteroidaceae*, and *Alloprevotella*, while elevating the production of SCFAs (acetate, propionate, and butyrate). Through the gut–retina axis, this multi-targeted strategy synergistically attenuated inflammation and oxidative stress, establishing a comprehensive defense mechanism against blue light-induced retinal degeneration.

## Figures and Tables

**Table 1:** Primers for qRT-PCR.

Gene	Primer Sequence
*Nrf2*	F:5′-TCAGCGACGGAAAGAGTATGA-3′ R:5′-CCACTGGTTTCTGACTGGATGT-3′
*Ho-1*	F:5′-CCAGCAACAAAGTGCAAGGT-3′ R:5′-CAGGAAACAACACCCACCAC-3′
*Nqo1*	F:5′-CACCACCTCCCATCCTTTCTT-3′ R:5′-GGTTTGCTGGTTGGTAATGGG-3′
*Gapdh*	F:5′-CTGACTTCAACAGCGACACC-3′ R:5′-CGCCAGACCCTGCACTTTTT-3′
*Zo-1*	F:5′-GCCGCTAAGAGCACAGCAA-3′ R:5′-GCCCTCCTTTTAACACATCAGA-3′
*Claudin*	F:5′-TGCAAAGTACCTGGTGGGAA-3′ R:5′-GCATCCTCCTAGCAACCGTC-3′
*Occludin*	F:5′-TACTGGTCTCTACGTGGATCAAT-3′ R:5′-TTCTTCGGGTTTTCACAGCAA-3′
*β-actin*	F:5′-ATCGCTGCGCTGGTCG-3′ R:5′-AGTCCTTCTGACCCATTCCC-3′

## Data Availability

The data underlying this article are available in the article and in its online Supplementary Materials. Further information pertaining to this research outcome can be sourced from Chen Liu (liuchenstc@163.com) in accordance with the reasonable requirements of the enquirer.

## Supplementary Materials

The following supporting information can be downloaded at https://www.mdpi.com/article/10.3390/nu17223517/s1.
